# Sequential Emergence and Wide Spread of Neutralization Escape Middle East Respiratory Syndrome Coronavirus Mutants, South Korea, 2015

**DOI:** 10.3201/eid2506.181722

**Published:** 2019-06

**Authors:** Yeon-Sook Kim, Abdimadiyeva Aigerim, Uni Park, Yuri Kim, Ji-Young Rhee, Jae-Phil Choi, Wan Beom Park, Sang Won Park, Yeonjae Kim, Dong-Gyun Lim, Kyung-Soo Inn, Eung-Soo Hwang, Myung-Sik Choi, Hyoung-Shik Shin, Nam-Hyuk Cho

**Affiliations:** Chungnam National University School of Medicine, Daejeon, South Korea (Y.-S. Kim);; Seoul National University College of Medicine, Seoul, South Korea (A. Aigerim, U. Park, Y. Kim, W.B. Park, S.W. Park, E.-S. Hwang, M.-S. Choi, N.-H. Cho);; Dankook University College of Medicine, Cheonan, South Korea (J.-Y. Rhee); Seoul Medical Center, Seoul (J.-P. Choi);; National Medical Center, Seoul (Y. Kim, D.-G. Lim, H.S. Shin); Kyung Hee University, Seoul (K.-S. Inn);; Seoul National University Medical Research Center and Bundang Hospital, Seoul (N.H. Cho)

**Keywords:** Middle East respiratory syndrome coronavirus, MERS-CoV, superspreading, spike, antibody neutralization, viruses, South Korea, respiratory infections, sequential emergence, zoonoses

## Abstract

The unexpectedly large outbreak of Middle East respiratory syndrome in South Korea in 2015 was initiated by an infected traveler and amplified by several “superspreading” events. Previously, we reported the emergence and spread of mutant Middle East respiratory syndrome coronavirus bearing spike mutations (I529T or D510G) with reduced affinity to human receptor CD26 during the outbreak. To assess the potential association of spike mutations with superspreading events, we collected virus genetic information reported during the outbreak and systemically analyzed the relationship of spike sequences and epidemiology. We found sequential emergence of the spike mutations in 2 superspreaders. In vivo virulence of the mutant viruses seems to decline in human patients, as assessed by fever duration in affected persons. In addition, neutralizing activity against these 2 mutant viruses in serum samples from mice immunized with wild-type spike antigen were gradually reduced, suggesting emergence and wide spread of neutralization escapers during the outbreak.

Middle East respiratory syndrome coronavirus (MERS-CoV) is a newly emerging zoonotic pathogen that causes an acute and fatal respiratory disease (*1*). The viral pathogen was first identified in September 2012 in an acute pneumonia patient in Saudi Arabia and has since been associated with 2,279 confirmed cases (with a death rate of ≈35.4%) in 27 countries as of March 2019 (https://www.who.int/emergencies/mers-cov). Although primary transmission of MERS-CoV to humans is linked to contact with dromedary camels, up to 50% of outbreak cases have been associated with human-to-human transmission, especially in healthcare settings (*1*). During May–July 2015, an unexpectedly large outbreak of MERS swept South Korea, resulting in 186 confirmed cases and 38 deaths (death rate 20.4%). This outbreak was initiated by an infected traveler from the Middle East region and amplified by several “superspreading” events (defined as >4 human-to-human transmissions) in healthcare settings (*2*). Three superspreaders (P001, P014, and P016) were epidemiologically linked to 73.1% of the human transmissions and infected 28, 85, and 23 subjects, respectively. Even though nosocomial superspreading might be facilitated by delayed diagnosis and poor infection control in healthcare facilities (*1*), the contribution of biologic factors, including host responses and virologic changes, has been poorly characterized. In addition, superspreading events continue to sporadically arise and lead to unexpectedly large outbreaks of MERS (*2*–*4*). Therefore, host–pathogen interactions driving virus evolution and human adaptation, which are potentially associated with rare superspreading events during host changes of the enzoonotic virus (*5*), need to be further investigated.

In previous studies, we reported the emergence and spread of mutant MERS-CoV bearing spike mutations (I529T or D510G) in receptor binding domain (RBD) with reduced affinity to human CD26 receptor during the South Korea outbreak (*6*). These unexpected findings suggest that MERS-CoV adaptation during human-to-human spread might be driven by host immunologic pressure, such as neutralizing antibodies (*7*–*9*), that result in impaired virus fitness and virulence, rather than positive selection for a better affinity to CD26. A recent report also showed that changes in D510G and I529T reduced spike protein binding to CD26 and diminished virus entry (*10*). 

To assess the potential contribution of new emerging mutations to superspreading events, we collected virus genetic information reported during the South Korea outbreak and systemically analyzed its variations, especially spike sequences, in relationship to individual disease severity and epidemiology. We also attempted to confirm whether the spike mutations affect virus dynamics in an in vitro infection model and virus escape from neutralizing antibody responses by using serum samples from mice immunized with wild-type spike antigen and from MERS patients in South Korea who had been infected with wild-type virus. Systemic overview of clinical and virologic data obtained during the transient but large outbreak driven by unexpected superspreading events among humans might provide novel insight into understanding the evolutionary pathways of the emerging coronavirus during animal-to-human transmission.

## Materials and Methods

### Study Design and Ethics

We collected genetic information on spike genes of MERS-CoV analyzed in the patients’ specimens from the National Center for Biotechnology Information (http://www.ncbi.nlm.nih.gov/) and a previous analysis by Park et al. (*11*). As of January 31, 2018, a total of 75 spike gene sequences from 48 patients in South Korea were available for analysis (Appendix Tables 1,2). Baseline characteristics of the patients are summarized (Table; Appendix Table 1). Clinical data and serum samples (from P002, P009, and P010) obtained from the MERS patients were used in this study. Experimental methods are described more fully in the Appendix.

**Table:** Baseline characteristics of MERS patients and associated MERS coronavirus spike genotypes identified from the 2015 MERS outbreak in South Korea.

Severity group	No. (%) patients		Patient age, y, mean + SD	No. associated spike genotypes				
	Men	Women		WT	I529T	D510G	WT–I529T	WT–I529T–D510G
I	3 (50.0)	3 (50.0)	54 + 13	1	2	1	1	1
II	9 (47.4)	10 (52.6)	46 + 11	0	10	2	6	1
III	8 (75.0)	4 (25.0)	48 + 12	3	4	0	4	1
IV	7 (63.6)	4 (36.4)	68 + 13	1	6	0	3	1

*MERS, Middle East respiratory syndrome; WT, wild-type.

Ethics approval was granted by the institutional review boards of Chungnam National University Hospital (approval no. CNUH2017–12–004), National Medical Center (approval no. H-1510–059–007), Seoul National University Hospital (approval nos. 1509–103–705 and 1511–117–723), Seoul National University Boramae Medical Center (approval no. 26–2016–8), Seoul Medical Center (approval no. Seoul 2015–12–102), and Dankook University Hospital (approval no. DKUH2016–02–014).

This study was conducted in accordance with the ethical standards laid down in the 1964 Declaration of Helsinki and all subsequent revisions. Animal experiments were approved by Seoul National University Institutional Animal Care and Use Committee (permit no. SNU-170828–1-2) and performed in strict accordance with the recommendations in the South Korea’s National Guideline for the Care and Use of Laboratory Animals.

## Results

**Figure 1:**
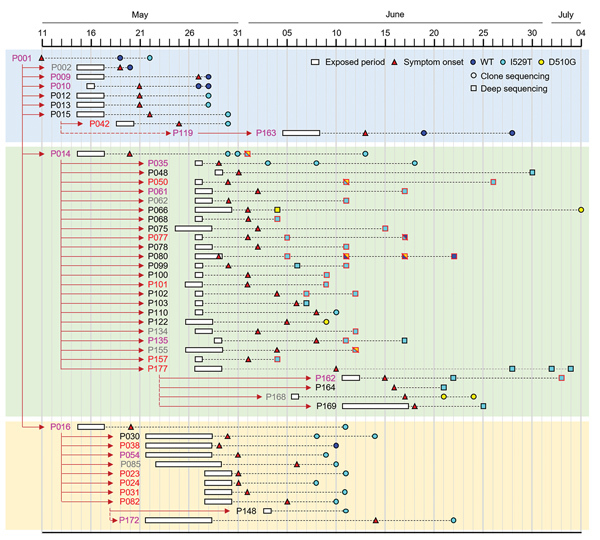
Emergence and spread of Middle East respiratory syndrome coronavirus (MERS-CoV) bearing the I529T or D510G mutation in the spike protein during the 2015 outbreak in South Korea. Transmission chain of infection and the timeline of potential virus exposure, symptom onset, date of specimen collection from patients, and identified mutation in the spike protein of MERS-CoV analyzed in this study. Case-patients’ IDs are colored on the basis of disease severity (gray, group I; black, group II; pink, group III; red, group IV). Spike sequences analyzed by targeted deep sequencing (*10*) are denoted as a square with black (single genotype) or red (mixed genotypes with wild-type) borderline. Others are marked as circles (direct sequencing). Detailed information on patients’ characteristics and their associated spike sequences of MERS-CoV are available in Appendix Tables 1 and 2). WT, wild-type.

We collected and analyzed genetic information of spike genes reported during the South Korea outbreak. All the information on the spike mutations in 48 patients and their sampling dates are summarized in Appendix Tables 1 and 2. The timelines of the detection of spike mutations are depicted in Figure 1. We focused on 2 mutations (D510G and I529T) in the RBD region because these 2 novel mutations substantially reduced MERS-CoV affinity to human receptor CD26 and were observed in multiple patients during the outbreak (*6*). In addition, no nonsynonymous spike mutation was consistently associated with the 2 spike mutations (Appendix Table 2). Wild-type MERS-CoV was detected in the index patient (P001) on May 19, 2015; however, the I529T mutation was observed 3 days later (May 22, 2015), suggesting that the I529T mutation emerged within 11 days after symptom onset. The potential change in spike gene is consistent with the fact that early contactors (P002, P009, and P010) with P001 also carried the wild-type virus, but most of the subsequent patients, including 2 superspreaders (P014 and P016), were infected with I529T mutant viruses. Moreover, the second mutation (D510G) was first detected in P014. The respiratory sample collected from P014 on June 1, 2015, included mixed spike sequences (wild-type, I529T, or D510G) and a rare double mutation (D510G–I529T) (*11*). Even though most of the patients infected by P014 carried MERS-CoV with the I529T mutation, the second mutant D510G was transiently or consistently observed in some of the tertiary and quaternary cases (P050, P066, P080, P122, P155, and P168) (*11*). These results indicate that P014 initially harbored mixed wild-type and I529T mutant viruses and generated the D510G mutation, but the I529T mutant virus dominated the exposure period. In addition, this patient spread both mutants simultaneously during subsequent human-to-human transmission. The D510G mutant was detected only in the patient group infected by P014 but not in those infected by P001 or P016, further indicating that P014 is the probable origin of the D510G mutation. The I529T mutant was initially dominant in 2 patients (P077 and P080), but wild-type MERS-CoV later overtook as the major population, suggesting a fluctuation of wild-type and mutant viruses in the hosts.

**Figure 2:**
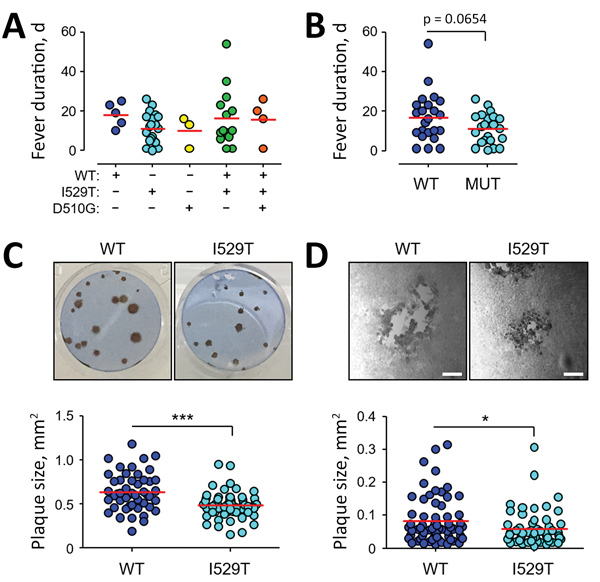
Effect of spike mutations in Middle East respiratory syndrome coronavirus (MERS-CoV) on fever duration and virus growth in vitro during the 2015 outbreak in South Korea. A) Fever duration of 48 patients for whom virus spike sequence information is available is presented depending on the associated spike genotypes. B) Fever duration of patient group associated with WT virus, including mixed infection (n = 23) and those infected only with either of the mutant viruses (n = 25). Mean value of each group is indicated by red lines. Baseline information of the patients and their associated MERS-CoV spike sequences are summarized in online Appendix Table 1. C, D) Distribution of viral plaque sizes in Vero cells (panel C: WT, n = 48; I529T, n = 58) or 293T–CD26 cells (panel D: WT, n = 65; I529T, n = 55) infected with MERS-CoV bearing WT or I529T mutant spike at 3 days after infection. Representative results of plaque assay are presented in the upper panels, and size distribution of viral plaques are plotted in the lower panel. Mean values are indicated by red lines. Significance was calculated by using a 2-tailed student’s *t*-test. WT, wild-type. ***p<0.001, *p<0.05. Scale bar indicates 100 μm.

Whether spike mutations of MERS-CoV are associated with disease morbidity has not yet been determined. We reviewed the clinical data of 48 patients for whom virus spike sequence information was available and classified them into 4 groups on the basis of disease severity and death rates during the MERS outbreak (Table; Appendix Table 1) (*12*). Group I includes 6 persons who were asymptomatic or had fever without pneumonia. Group II includes 19 patients who had mild pneumonia without hypoxemia. Twelve persons who recovered from more prolonged and severe pneumonia are classified as group III. Group III subjects experienced hypoxemia and were treated with oxygen during hospitalization. Eleven patients who died from acute respiratory distress syndrome are classified as group IV. Baseline characteristics of the patients, including exposure period, date of symptom onset, and fever duration, are summarized (Figure 1; Appendix Table 1). Initially, we assessed the potential association of spike mutations with disease severity (Table), but could not observe any statistically significant association of MERS severity with spike mutations or mixed spike genotype infection. However, the average days of fever duration in MERS patients was generally longer in cases associated with wild-type virus (mean + SD, 18 + 6 days) or mixed infection including wild-type (mean + SD, 16 + 14 days) compared with fevers in patients infected only with I520T (mean + SD, 11 + 8 days) or D510G (mean + SD, 10 + 8) mutant (Figure 2, panel A). Even though differences in fever duration between the patient group associated with wild-type virus, including mixed infection (17 + 12 days), and those infected only with either of the mutant viruses (mean + SD, 11 + 7 days) are statistically marginal (p = 0.0654) (Figure 2, panel B), potentially because of limited data, the reduction of fever duration in patients associated only with the primary and secondary mutations seems to be consistent.

Because the disease severity and case-fatality rate of MERS is associated with viral load (*12*), we next examined whether mutant viruses with reduced affinity to human CD26 receptor had also reduced cell-to-cell spread, virus growth, or both. We performed plaque-forming assay in vitro and compared viral plaques between wild-type and I529T MERS-CoV. The average size of viral plaques formed by I529T mutant MERS-CoV (mean + SD, 0.49 + 0.15 mm^2^) was significantly smaller (≈23%) than that of wild-type virus (mean + SD, 0.64 + 0.21 mm^2^) in Vero E6 cells (Figure 2, panel C). It was consistently observed in a human embryonic kidney cell line, 293T cells overexpressing human CD26 (293T–CD26) (Figure 2, panel D). The average size of plaques induced by I529T mutant (mean + SD, 0.08 + 0.07 mm^2^) was also smaller (≈25%) than that of wild-type MERS-CoV (mean + SD, 0.06 + 0.05 mm^2^) in 293T–CD26 cells. These results clearly indicate that spike mutation generated during the South Korea outbreak have reduced transmissibility, reduced growth rate, or both in an in vitro infection model.

**Figure 3:**
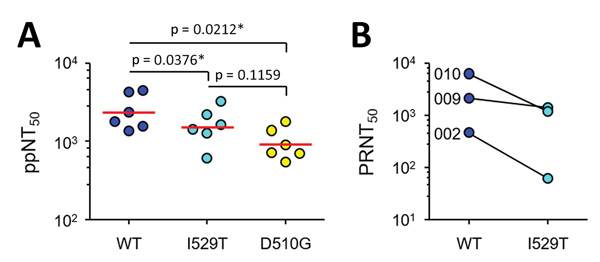
Increased resistance of Middle East respiratory syndrome coronavirus (MERS-CoV) against antibody-mediated neutralization by spike mutations during the 2015 outbreak in South Korea. A) Neutralizing activity of serum samples against lentiviruses bearing WT and mutant spikes. 50% pseudoparticle neutralization test titers against lentiviruses bearing WT or mutant spikes (I529T or D510G) in serum samples from mice (n = 6) immunized with WT spike antigen are plotted. Mean values are indicated by red lines. Statistical significance was calculated by using analysis of variance with Newman–Keuls post *t*-test correction. *p<0.05. B) Neutralization activity against MERS-CoV bearing WT or I529T mutant spike mutation in serum samples from 3 recovered patients (P002, P009, and P010) who carried only WT MERS-CoV. ppNT_50_, 50% pseudoparticle neutralization test; PRNT_50_, 50% plaque reduction neutralization test; WT, wild-type.

To examine whether the spike mutations affect sensitivity to the neutralizing activity of antibodies against wild-type spike, we investigated whether the antibodies generated in mice immunized with wild-type spike antigen are able to neutralize the spike mutant viruses as efficiently as wild-type virus. We performed a 50% pseudoparticle neutralization test assay on wild-type and mutant spike-pseudotyped lentiviruses and then compared their neutralizing efficacy. Average titers of serum samples from the immunized mice showed gradually decreased neutralization of I529T (mean + SD, 1,727 + 897) or D510G mutant viruses (1,009 + 482) than wild-type virus (mean + SD, 2,629 + 1,384) (Figure 3, panel A), demonstrating that the mutant viruses are neutralization escapers. Finally, we measured the neutralizing antibody titers (using a 50% plaque reduction neutralization test [PRNT_50_]) against MERS-CoV bearing wild-type and I529T mutant in serum samples from the 3 recovered patients (P002, P009, and P010) who carried only wild-type MERS-CoV. Neutralizing efficacy (PRNT_50_ titers) of the serum samples against wild-type MERS-CoV were consistently higher than those against I529T mutant MERS-CoV (Figure 3, panel B). Average PRNT_50_ titers of the 3 serum samples against wild-type (mean + SD, 2,943 + 2,994) were 3.3-fold higher than those against I529T mutant MERS-CoV (mean + SD, 888 + 723), indicating that I529T mutant MERS-CoV escapes better from neutralizing antibodies generated by infection with wild-type virus. PRNT_50_ titers of serum samples from P009 and P010, who had more severe disease, were higher than those of P002, who had only mild symptoms.

## Discussion

Even though multiple environmental and behavioral factors might be associated with the zoonotic coronavirus outbreaks in human populations, the advent of “superspreaders” that cause a large number of new infections during the early stage of an outbreak might play a critical amplifying role (*13*). Such superspreading persons have been well documented in a previous severe acute respiratory syndrome (SARS) epidemic and recent ongoing MERS outbreaks (*13*). Although the underlying biologic causes and determinants of superspreading are still poorly understood, superspreading events of zoonotic coronaviruses might be related to higher levels of virus shedding for prolonged periods of uncontrolled exposure to susceptible contacts early in the outbreak, before the need for infection control is appreciated (*5*). Our study systemically assesses MERS-CoV genetic changes during the 2015 MERS outbreak in Korea and highlights potential biologic factors associated with the short-term evolutionary pathway of zoonotic coronavirus adapting to the human population in the context of superspreading events.

Microevolution toward reduced host affinity of MERS-CoV might be associated with host antibody responses. In this study, we showed that antibodies generated in immunized mice with wild-type spike antigen or in human patients who had recovered from wild-type MERS-CoV infection were generally less efficient at neutralizing the mutant virus bearing I529T or D510G spike mutations than wild-type virus (Figure 3). In addition, in vitro virus growth of I529T mutant MERS-CoV, its cell-to-cell spread, or both are significantly reduced compared with that of wild-type virus (Figure 2, panel C and D). Given that there are only 2 amino acid differences in spike RBD (I529T and V534L) and 5 nonsynonymous nucleotide changes throughout the whole virus genome (GenBank accession no. KT029139.1 for wild-type and KT868873.1 for I529T mutant) between the wild-type and I529T mutant isolates from Korea used in this study, the difference in plaque sizes is primarily attributable to the spike mutation (Appendix Table 3). A recent study demonstrated that changes D510G or I529T increased resistance of spike protein–driven entry to neutralization by monoclonal antibodies and serum from MERS patients (*10*). The study also confirmed that changes D510G or I529T reduced spike protein binding to CD26 but showed that this reduction only translates into diminished virus entry when expression of CD26 on target cells is low (*10*). Neither mutation modulated spike protein binding to sialic acids, spike protein activation by host cell proteases, or inhibition of spike protein–driven entry by interferon-induced transmembrane proteins (*10*). In this study, however, we consistently observed that the average size of viral plaques formed by the mutant MERS-CoV bearing I529T mutation was significantly reduced in 2 types of permissive cells, Vero E6 and HEK293T-CD26 cells expressing high levels of CD26 (*14*,*15*), compared with that of wild-type MERS-CoV (Figure 2C and 2D). 

Because a previous study employed a pseudotyped vesicular stomatitis virus system bearing wild-type or mutant MERS-CoV spike proteins (*10*), our current result using MERS-CoV isolated from human patients might reflect more intrinsic characteristics of spike protein in the context of the natural form of MERS-CoV. Further studies are needed to characterize the effect of the spike mutations on virus growth or spreadin in vitro and in vivo infection systems.

The emergence of spike mutations that affect affinity to host receptors might be a critical cause of superspreading events. A similar observation was made regarding the 2002–2003 SARS epidemic (*13*). However, those mutations generally enhanced the affinity of spike to human receptor ACE2 and was detected from the beginning of human-to-human transmission (*16*–*19*). Both the 2002–2003 SARS epidemic and the 2015 MERS outbreak in South Korea were associated virus mutations that change affinity to human receptors, but in opposing ways: SARS-CoV acquires mutations to replicate more efficiently and become more pathogenic (*13*), whereas MERS-CoV mutants, which have less affinity to receptors on human cells and potentially less pathogenicity, emerged in superspreaders during the outbreak in South Korea. 

A recent study reported that both MERS-CoV and SARS-CoV spike trimers have inherently flexible RBD structure with 2 states (buried flat or exposed in a standing position) as observed by cryoelectron microscopic analysis (*20*). That study proposed that 1 CD26 molecule might cross-link 2 MERS-CoV spike trimers by binding to standing RBDs, 1 from each trimer, whereas the monomeric ACE2 receptor will bind to the SARS-CoV spike trimer with 1 receptor to 1 spike trimer, enabling MERS-CoV to have higher avidity to receptor binding than SARS-CoV (*20*). Inherent differences in avidity to the cellular receptors of spike trimers might partially explain why these 2 animal coronaviruses evolved in opposing directions in terms of receptor affinity when they switch hosts to the human population; spike mutations with higher affinity might be the only way to ensure successful human-to-human transmission in SARS-CoV with intrinsic lower avidity, whereas the higher spike avidity of MERS-CoV might afford the virus to have spike mutations with lower affinity to human receptors for increased antibody escape. Therefore, a vaccine immunogen designed to broadly raise neutralizing antibodies would preferably target the conserved and surface exposed stem region rather than RBD (*20*). Recently, a study showed that single mutations can make influenza virus completely resistant to both narrow strain-specific antibodies and a broad antibody that targets residues in hemagglutinin RBD, whereas broad antibodies to hemagglutinin’s stalk are more resistant to virus escape through single mutations (*21*).

The microevolution of spike genes in MERS-CoV toward reduced human affinity but enhanced escape from neutralizing antibodies in a single patient might increase the probability of a spreading event by extending the virus replication period in the host. The 3 superspreaders in South Korea (P001, P014, and P016) belong to group III and had severe and prolonged viral pneumonia (*2*). The group III patients produced a significantly higher number of viruses in their respiratory secretions for longer periods than patients with milder cases (*12*,*22*). The superspreaders in South Korea were suspected to produce MERS-CoV with high copy numbers (10^8^–10^9^ copies/mL) in their respiratory secretions during the early phase of MERS symptom development (*12*,*22*,*23*) and exposure to susceptible contacts for 9–11 days (*2*). They were also positive for virus in their respiratory secretions for prolonged periods (44, 30, and 27 days, respectively, after symptom onset) even after isolation (*12*,*22*,*24*). In addition, a new spike mutant emerged in the index patient (P001) and P014, who combined infected >80 patients, within 2 weeks after symptom onset, such that they harbored mixed infection with wild-type and spike mutant viruses. Consequently, P001 and P014 spread mixed viruses during the early phase of the outbreak (Figure 1) (*11*). 

Mixed infection with wild-type and spike mutant viruses was confirmed in the majority of the tertiary case-patients infected by P014 by targeted deep sequencing, and the intrapatient heterogeneity of MERS-CoVs was the highest in superspreader specimens (*11*). Although the frequency of D510G and I529T varied greatly among specimens analyzed using targeted deep sequencing, the combined frequency of the single mutants was consistently high (≈88% on average), whereas the frequency of the wild-type was low (≈7% on average), supporting the hypothesis that selective pressure exerted by host neutralizing antibody responses played a critical role in shaping genetic variants (*11*). 

Fluctuations of wild-type population with higher affinity to host and mutant viruses that can escape neutralizing antibodies in a single person might facilitate sustained virus replication with higher loads. Wild-type virus was dominant in several tertiary patients (e.g., P077 and P080) at the later stage of infection, although mutant virus was the major population in the earlier stage (Figure 1). P077 had hypotension, chronic respiratory disease, and pancreatitis. P080 had lymphoma and respiratory illness before infection by P014. P077 died 13 days after symptom onset, and P080 died of lymphoma 174 days after symptom onset. In both cases, wild-type and mutant viruses were detected initially (*11*), suggesting a mixed infection from P014, and the patient might have suffered from immunosuppression upon virus infection either by initial high viral load (in the case of P077), as observed in other fatal cases (*12*), or by previous cancer treatment (in the case of P080). Failing adaptive immunity in these patients might provide a specific environment that allowed wild-type virus with higher affinity to host to resurge among the mixed population in the later stage of infection. Indeed, serial samples from P077 and P080 show a substantial decrease in normalized leukocyte count and a simultaneous increase in the frequency of the wild-type allele (*11*). Again, these results suggest that the selection pressure exerted by the host immune response might favor variants with reduced affinity to the host receptor, but wild-type virus with high affinity is dominant when immune pressure is reduced. 

Taken together, the emergence of antibody escaping mutants under mounting immunologic pressure in a host might ensure sustained virus replication, higher virus shedding into respiratory secretions for longer periods, and delay in antigen-specific immunity, thereby increasing the probability of a patient becoming a superspreader. Nevertheless, this evolutionary pathway of coronaviruses during human-to-human spread might result in serial decrease of host affinity and pathogenicity, as well as milder respiratory symptoms, if their transmission in the human population is not properly restricted at the initial outbreak stage.

Appendix Additional information regarding the sequential emergence and wide spread of neutralization escape MERS-CoV mutants, South Korea, 2015.
