# Supplementary material for: Sequential Emergence and Wide Spread of Neutralization Escape Middle East Respiratory Syndrome Coronavirus Mutants, South Korea, 2015
Source: Emerg Infect Dis. 2019 Jun;25(6):1161–8. doi: 10.3201/eid2506.181722 (PMC6537729; doi:10.3201/eid2506.181722)
Supplement: Appendix — Additional information regarding the sequential emergence and wide spread of neutralization escape MERS-CoV mutants, South Korea, 2015. [file 18-1722-Techapp-s1.pdf]

Article DOI: https://doi.org/10.3201/eid2506.181722

Sequential Emergence and Wide Spread of Neutralization Escape Middle East Respiratory Syndrome Coronavirus Mutants, South Korea, 2015

Appendix

Materials and Methods

Middle East Respiratory Syndrome Coronavirus Culture and Plaque Assay

Wild-type Middle East respiratory syndrome coronavirus (MERS-CoV) or I529T mutant MERS-CoV isolated from patients in South Korea (GenBank accession nos. KT029139.1 for wild type and KT868873.1 for I529T mutant) were cultured in a 24-well plate containing a monolayer of Vero E6 cells or 293T cells stably expressing CD26 (*1*). After 1 h incubation at 37°C, viral supernatant was removed and cells were overlaid with 1 mL of 1% methylcellulose in Dulbecco modified Eagle medium, including 10% fetal bovine serum. Plates were incubated for 3 d at 37°C, and then cells were fixed with 4% paraformaldehyde and 100% methanol. The MERS-CoV plaques were detected using rabbit anti-MERS-CoV N protein antibody (Sino Biologic Inc.) and goat anti-rabbit IgG secondary antibody conjugated with horseradish peroxidase (Invitrogen; https://www.thermofisher.com/us/en/home/brands/invitrogen.html). Viral plaques were visualized by incubation with 0.05% 3′3-diaminobenzidine tetrahydrochloride and 0.01% hydrogen peroxide in 50 mmol/L Tris-HCl (pH 8.0). Cellular layers were counterstained with trypan blue dye.

Neutralizing Antibody Assays

Pseudotyped lentiviruses with wild type or mutant spikes of MERS-CoV were generated from 293T cells (Invitrogen) by cotransfection of human immunodeficiency virus backbone plasmids expressing firefly luciferase as previously described (*1*). We used the packaging plasmids, pLP1, pLP2, and pLP/VSV-G (Invitrogen) and pLVX-Luc-IRES-ZsGreen1 (Clontech; https://www.takarabio.com). For spike protein pseudotyping, condon-optimized cDNA of the

spike gene (Sino Biological; https://www.sinobiological.com) was cloned into pcDNA3 after deleting an ER/Golgi retention motif and an endosomal recycling motif from the cytoplasmic tail (*2*) for transfection instead of pLP/VSV-G. A plasmid carrying the gene encoding the I529T or D510G mutation in spike protein was generated by using the QuikChange kit (Stratagene; http://go.strategene.org/genetic-analysis) based on the wild-type construct, and the point mutation was confirmed by sequencing. Viral supernatants were harvested 48 h after transfection and normalized by p24 ELISA kit (Clontech) before infecting 293T cells expressing human CD26 (293T-CD26) (*1*).

To assess the neutralizing activity by spike pseudoparticle neutralization assay (*3*), pseudoviruses (0.1 multiplicity of infection) were preincubated with serially diluted serum samples from mice immunized three times with wild-type spike antigen (Sino Biologic Inc.) at 4°C for 1 h. Subsequently, the infected 293T-CD26 cells were lysed 48 h after infection, and the efficiency of viral entry was measured by comparing luciferase activity. The relative luciferase activity in cell lysates was measured using a luciferase assay kit (Promega; https://www.promega.com) and Infinite 200 PRO microplate reader (Tecan; https://lifesciences.tecan.com). Neutralization titers of collected serum samples against MERS-CoV were also determined by a plaque reduction neutralization titer assay. Each serum sample collected from convalescent-phase patient was serially diluted and incubated with wild-type MERS-CoV or I529T mutant MERS-CoV (0.004 multiplicity of infection) for 1 h at 4°C. The viruses were then added to a 24-well plate containing a monolayer of Vero E6 cells in duplicate. After 1 h incubation at 37°C, viral supernatant was removed and cells were overlaid with 1 mL of 1% methylcellulose in Dulbecco modified Eagle medium including 10% fetal bovine serum. Viral plaques were visualized as described above. The percentage of plaque reduction was calculated as [(no. of plaques without antibody) – (no. of plaques with antibody)] / (no. of plaques without antibody) x 100. The 50% pseudoparticle neutralization assay nd 50% plaque reduction neutralization titers were calculated by a nonlinear regression analysis (log[inhibitor] versus normalized response method) embedded in GraphPad Prism Software v5.01 (GraphPad Software; https://www.graphpad.com).

Statistical Analysis

Data were analyzed using GraphPad Prism Software. Statistical analysis was performed using a 2-tailed Student *t*-test or one-way analysis of variance, followed by the Newman–Keuls

t-test for comparisons of values among different groups. $p<0.05$ was considered statistically significant.

References

1. Kim Y, Cheon S, Min CK, Sohn KM, Kang YJ, Cha YJ, et al. Spread of mutant Middle East respiratory syndrome coronavirus with reduced affinity to human CD26 during the South Korean outbreak. MBio. 2016;7:e00019. PubMed http://dx.doi.org/10.1128/mBio.00019-16

2. Min CK, Cheon S, Ha NY, Sohn KM, Kim Y, Aigerim A, et al. Comparative and kinetic analysis of viral shedding and immunological responses in MERS patients representing a broad spectrum of disease severity. Sci Rep. 2016;6:25359. PubMed http://dx.doi.org/10.1038/srep25359

3. Wong G, Liu W, Liu Y, Zhou B, Bi Y, Gao GF. MERS, SARS, and Ebola: the role of super-spreaders in infectious disease. Cell Host Microbe. 2015;18:398–401. PubMed http://dx.doi.org/10.1016/j.chom.2015.09.013

4. Park D, Huh HJ, Kim YJ, Son DS, Jeon HJ, Im EH, et al. Analysis of intrapatient heterogeneity uncovers the microevolution of Middle East respiratory syndrome coronavirus. Cold Spring Harb Mol Case Stud. 2016;2:a001214. PubMed http://dx.doi.org/10.1101/mcs.a001214

Appendix Table 1. Baseline characteristics of Middle East respiratory syndrome patients and spike mutations associated with the patients*

Patient ID	Sex	Age, y	Plausible source	Clinical severity	Fever duration, d	Sampling date	GenBank accession no.	MERS-CoV spike mutations‡
P001	M	68		3	54	2015 May 19	KT182958.1	WT
						2015 May 22	KT326819.1	I529T
P002	F	63	1	1	10	2015 May 20	KT029139.1	WT
P009	M	55	1	3	25	2015 May 28	KT182953.1	WT
P010	M	44	1	3	14	2015 May 27	KT006149.2	WT
						2015 May 28	KT036372.1	WT
P012	F	49	1	2	9	2015 May 28	KT182954.1	I529T
P013	M	49	1	2	1	2015 May 28	KT182955.1	I529T
P014	M	35	1	3	16	2015 May 30	KX034093.1	I529T
						2015 May 31	KT374052.1	I529T
						2015 Jun 1	†	WT/I529T/D510G
						2015 Jun 13	KT374053.1	I529T
P015	M	35	1	2	7	2015 May 30	KT182956.1	I529T
P016	M	41	1	3	13	2015 Jun 11	KT868865.1	I529T
P023	M	73	16	4	5	2015 Jun 11	KT868866.1	I529T
P024	M	78	16	4	0	2015 Jun 8	KT868867.1	I529T
P030	M	60	16	2	23	2015 Jun 8	KT868868.1	I529T
P031	M	69	16	4	17	2015 Jun 11	KT868869.1	I529T
P035	M	38	14	3	?	2015 Jun 3	KT374054.1	I529T
						2015 Jun 8	KU308549.1	
						2015 Jun 18	KT374055.1	
P038	M	49	16	4	19	2015 Jun 10	KT868870.1	WT
P042	F	54	1-11	4	?	2015 May 30	KT182957.1	I529T
P048	M	38	14	2	12	2015 May 30	†	I529T
P050	F	80	14	4	26	2015 Jun 11	†	WT/I529T/D510G
						2015 Jun 11	KX034094.1	D510G
						2015 Jun 26	†	I529T
P054	F	63	16	3	16	2015 Jun 9	KT868871.1	I529T
P061	M	55	14	3	27	2015 Jun 17	†	I529T
P062	M	51	14	1	5	2015 Jun 11	†	I529T
P066	F	42	14	2	16	2015 Jun 4	†	D510G
						2015 Jul 4	KX034095.1	D510G
P068	F	55	14	2	6	2015 Jun 4	†	I529T
P075	M	62	14	2	1	2015 Jun 15	†	I529T
P077	M	63	14	4	10	2015 Jun 5	†	I529T
						2015 Jun 17	†	WT/I529T
						2015 Jun 17	KX034096.1	I529T
P078	F	41	14	2	9	2015 Jun 11	†	I529T
P080	M	34	14	2	20	2015 Jun 5	†	I529T
						2015 Jun 11	†	WT/D510G
						2015 Jun 17	†	WT/D510G
						2015 Jun 17	KX034097.1	D510G
						2015 Jun 22	†	WT
P082	F	83	16	4	13	2015 Jun 10	KT868872.1	I529T
P085	F	66	16	1	1	2015 Jun 10	KT868873.1	I529T
P099	M	48	14	2	9	2015 Jun 6	†	I529T
						2015 Jun 11	†	I529T
P100	F	32	14	2	10	2015 Jun 9	†	I529T
P101	M	85	14	4	20	2015 Jun 9	†	I529T
P102	F	48	14	2	7	2015 Jun 7	†	I529T
						2015 Jun 12	†	I529T
P103	M	66	14	2	4	2015 Jun 7	†	I529T
P110	F	57	14	2	20	2015 Jun 11	KT868874.1	I529T
P122	F	55	14	2	13	2015 Jun 10	KT868875.1	D510G
P134	F	68	14	1	1	2015 Jun 12	†	I529T
P135	M	33	14	3	23	2015 Jun 11	†	I529T
						2015 Jun 17	†	I529T
P148	F	39	16-36	2	6	2015 Jun 11	KT868876.1	I529T
P155	F	42	14	1	1	2015 Jun 12	†	WT/I529T/D510G
P157	M	60	14	4	35	2015 Jun 22	†	I529T
P162	M	33	14-?	3	18	2015 Jun 22	†	I529T
						2015 Jun 22	KX034098.1	I529T
						2015 Jul 1	†	I529T
P163	F	52	119	3	23	2015 Jun 19	KT374051.1	WT
						2015 Jun 29	KT374050.1	WT
P164	F	35	14-?	2	11	2015 Jun 21	†	I529T

Patient ID	Sex	Age, y	Plausible source	Clinical severity	Fever duration, d	Sampling date	GenBank accession no.	MERS-CoV spike mutations‡
P168	M	36	14-76	1	1	2015 Jun 21	KT374056.1	D510G
						2015 Jun 24	KT374057.1	D510G
P169	M	33	14-135	2	18	2015 Jun 26	†	I529T
						2015 Jun 26	KX034099.1	I529T
P172	F	61	16-?	3	26	2015 Jun 22	KT868877.1	I529T
P177	F	49	14	4	17	2015 Jun 28	†	I529T
						2015 Jul 1	†	I529T
						2015 Jul 3	†	I529T
						2015 Jul 3	KX034100.1	I529T

*ID, identification; MERS-CoV, Middle East respiratory syndrome coronavirus.
†Park et al. (*4*).
‡Spike sequences with mixed genotypes including wild type or indicated mutants were labeled as yellow-background or single genotype as gray-background in samples of targeted deep sequencing. Dominant amino acid sequences, occupying more than 50% in targeted deep sequencing were indicated.

Appendix Table 2. Summary of nonsynonymous mutations observed in spike sequences reported during the outbreak in South Korea*

Patient ID	Isolation date	GenBank accession no.	Nonsynonymous spike mutations													
			H91Y	R301C	Y351H	D510G	I529T	V534L	R529H	V718I	Q1020R	Q1056R	A1193E	V1209A	W1300#	P1347L
1	2015 May 19	KT182958.1	–	–	–	–	–	–	–	–	–	–	–	–	–	–
	2015 May 22	KT326819.1	–	–	–	–	+	–	–	–	+	–	–	–	–	–
2	2015 May 20	KT029139.1	–	–	–	–	–	+	–	–	+	–	–	–	–	–
9	2015 May 28	KT182953.1	–	–	–	–	–	–	–	–	+	–	–	–	–	–
10	2015 May 27	KT006149.2	–	–	–	–	–	–	–	–	+	–	+	+	–	–
	2015 May 28	KT036372.1	–	–	–	–	–	–	–	–	+	–	–	–	–	–
12	2015 May 28	KT182954.1	–	–	–	–	+	–	–	–	+	–	–	–	–	–
13	2015 May 28	KT182955.1	–	–	–	–	+	–	–	–	+	–	–	–	–	–
14	2015 May 30	KX034093.1	+	–	–	–	+	–	–	–	+	–	–	–	–	–
	2015 May 31	KT374052.1	–	–	–	–	+	–	–	–	+	–	–	–	–	–
	2015 Jun 13	KT374053.1	–	–	–	–	+	–	–	–	+	–	–	–	–	–
15	2015 May 30	KT182956.1	–	–	–	–	+	–	+	–	+	–	–	–	–	–
16	2015 Jun 11	KT868865.1	–	–	–	–	+	–	–	–	+	–	–	–	–	–
23	2015 Jun 11	KT868866.1	–	–	–	–	+	–	–	–	+	–	–	–	–	–
24	2015 Jun 8	KT868867.1	–	–	–	–	+	–	–	–	+	–	–	–	–	–
30	2015 Jun 8	KT868868.1	–	–	–	–	+	–	–	–	+	–	–	–	–	–
31	2015 Jun 11	KT868869.1	–	–	–	–	+	–	–	–	+	–	–	–	–	–
35	2015 Jun 3	KT374054.1	–	–	–	–	+	–	–	–	+	–	–	–	–	–
	2015 Jun 8	KU308549.1	–	–	–	–	+	–	–	–	+	–	–	–	–	–
	2015 Jun 18	KT374055.1	–	–	–	–	+	–	–	–	+	–	–	–	–	–
38	2015 Jun 10	KT868870.1	–	–	–	–	–	–	–	–	+	–	–	–	–	–
42	2015 May 30	KT182957.1	–	–	–	–	+	–	+	–	+	–	–	–	–	–
50	2015 Jun 11	KX034094.1	–	–	–	+	–	–	–	–	+	–	–	–	–	–
54	2015 Jun 9	KT868871.1	–	–	–	–	+	–	–	–	+	–	–	–	W/#†	–
66	2015 Jul 4	KX034095.1	–	–	–	+	–	–	–	–	+	–	–	–	–	–
77	2015 Jun 17	KX034096.1	–	–	–	–	+	–	–	–	+	–	–	–	–	–
80	2015 Jun 17	KX034097.1	–	+	–	+	–	–	–	–	+	–	–	–	–	–
82	2015 Jun 10	KT868872.1	–	–	–	–	+	–	–	+	+	–	–	–	–	–
85	2015 Jun 10	KT868873.1	–	–	–	–	+	–	–	–	+	–	–	–	–	–
110	2015 Jun 11	KT868874.1	–	–	–	–	+	–	–	–	+	–	–	–	–	–
122	2015 Jun 10	KT868875.1	–	–	–	+	–	–	–	–	+	–	–	–	–	–
148	2015 Jun 11	KT868876.1	–	–	–	–	+	–	–	+	+	–	–	–	–	–
162	2015 Jun 22	KX034098.1	–	–	–	–	+	–	–	–	+	–	–	–	–	–
163	2015 Jun 19	KT374051.1	–	–	–	–	–	–	–	–	+	+	–	–	–	–
	2015 Jun 29	KT374050.1	–	–	–	–	–	–	–	–	+	+	–	–	–	–
168	2015 Jun 21	KT374056.1	+	–	–	+	–	–	–	–	+	–	–	–	–	–
	2015 Jun 24	KT374057.1	+	–	–	+	–	–	–	–	+	–	–	–	–	+
169	2015 Jun 26	KX034099.1	–	–	–	–	+	–	–	–	+	–	–	–	–	–
172	2015 Jun 22	KT868877.1	–	–	Y/H†	–	+	–	–	–	+	–	–	–	–	–
177	2015 Jul 3	KX034100.1	–	–	–	–	+	–	–	–	+	–	–	–	–	–

*Nonsynonymous mutations observed by comparative analysis with the first isolate from P001. ID, identification; +, positive; –, negative. .

†Mixed sequences.

Appendix Table 3. Nucleotide sequence differences oberved in wild type and I529T mutant Middle East respiratory syndrome coronavirus isolates used in this study*

Nucleotide position	WT (KT029139.1)	I529T (KT868873.1)	ORF**	Amino acid mutation
19075	G	A	ORF1	NS
23041	T	C	S	I529T
23043	C	G	S	V534L
23303	T	C	S	NS
24383	C	T	S	NS
25968	T	A	ORF4a	NS
26109	T	C	ORF4a	NS

*NS, nonsynonymous; ORF, open reading frame; S, spike; WT, wild-type.
